# Bacteriocin-mediated intraspecies competition driven by acquired Bac41 operon in epidemic *Enterococcus faecalis* ST179

**DOI:** 10.1128/spectrum.03982-25

**Published:** 2026-08-06

**Authors:** Jinglin Yue, Tingzhu Yao, Nan Chen, Hengkun Wei, Aoze Cheng, Chengbo Rong, Huizhu Wang, Hui Li, Jingyuan Liu, Mingxi Hua, Chen Chen

**Affiliations:** 1 Biomedical Innovation Center, Beijing Shijitan Hospital, Capital Medical University 662230, Beijing, China; 2 Beijing Key Laboratory for Therapeutic Cancer Vaccines, Beijing, China; 3 Department of Blood Transfusion, Beijing Anzhen Hospital, Capital Medical University 12667, Beijing, China; 4 Department of Critical Care Medicine, Beijing Ditan Hospital, Capital Medical University 12638, Beijing, China; 5 Department of Stomatology, Beijing Shijitan Hospital, Capital Medical University 117968, Beijing, China; Institut National de Santé Publique du Québec, Sainte-Anne-de-Bellevue, Québec, Canada

**Keywords:** *Enterococcus faecalis*, intraspecies competition, bacteriocin, epidemic clone

## Abstract

*Enterococcus faecalis* is a common gut commensal and an opportunistic pathogen causing hospital-acquired infections. Despite its clinical importance, comprehensive global genomic and epidemiological data remain limited. Here, we analyzed 5,895 *E. faecalis* genomes collected between 2000 and 2020 and identified ST179, a human-derived single-operon variant of the high-risk CC16 clonal complex, as an emerging epidemic clone in China. Spot-killing assays revealed that ST179 strongly inhibited other clinical *E. faecalis* sequence types. Biochemical purification and proteomic analyses identified BacL1 as a key effector associated with this species-specific antibacterial activity. Functional assays confirmed its inhibitory phenotype, providing ST179 with a lineage-specific, bacteriocin-mediated competitive advantage. The high prevalence of the Bac41 operon likely contributed to the epidemiological success and ecological fitness of ST179. These findings highlight the role of bacteriocin-mediated intraspecies competition in shaping *E. faecalis* population dynamics and suggest that ST179 might become an emerging high-risk lineage in China.

**IMPORTANCE:**

*Enterococcus faecalis* is a common gut bacterium and an opportunistic pathogen. We identify ST179 as an emerging epidemic clone in China and show that it outcompetes other strains via the bacteriocin Bac41. This competitive advantage helps explain its rapid spread. Our findings highlight how bacterial competition shapes population dynamics and provide insights into the emergence of high-risk *E. faecali*s lineages, informing strategies for monitoring and infection control.

## INTRODUCTION

*Enterococcus faecalis* is a common commensal of the human gastrointestinal tract but also a major opportunistic pathogen responsible for hospital-acquired infections, including endocarditis, bacteremia, and urinary tract infections (1–3). Despite its clinical relevance, comprehensive global epidemiological and genomic data for *E. faecalis* remain limited. Most genomic surveillance efforts have concentrated on Europe and North America, leaving large-scale, longitudinal data sets from other regions scarce, particularly China (4, 5). Moreover, previous studies have focused predominantly on vancomycin-resistant enterococci, whereas the epidemiology and genomic features of vancomycin-susceptible *E. faecalis* (VSE) remain insufficiently characterized, even though VSE strains continue to impose a substantial clinical burden (6, 7).

*E. faecalis* exhibits striking genomic plasticity, enabling rapid acquisition of mobile genetic elements and other adaptive traits (3, 8). As a result, dominant epidemic sequence types (STs) can shift across time and geography, although the ecological drivers of these clonal turnovers remain poorly understood (3, 9). Recent genomic surveillance has highlighted ST179, a human-derived single-operon variant of the high-risk CC16 clonal complex, as a recurrent lineage in multiple regions, particularly within China (9–17). First reported in 2009–2010, ST179 has since been repeatedly detected in regional and global surveys conducted in 2013, 2017, and 2020, underscoring its growing clinical significance (9, 10, 18–20).

Although prior research has primarily examined virulence factors and antimicrobial resistance within well-known high-risk clonal complexes such as CC2, CC9, CC87, and CC16, the ecological mechanisms underlying the emergence and spread of *E. faecalis* lineages remain underexplored (3, 21). In particular, the role of intraspecies competitive interactions has received limited attention. Bacteriocins, antimicrobial peptides that target closely related strains, are critical mediators of clonal competition and replacement in *E. faecalis* (22, 23). While several bacteriocins have been characterized, their precise contributions to shaping clonal dynamics have not been systematically investigated (24, 25). In enterococci, bacteriolytic systems such as enterolysin A and bacteriocin 41 (Bac41) have been identified, both of which contribute to inter- and intraspecies antagonistic interactions (26–28). However, how these systems influence competitive interactions at the clonal level remains poorly understood.

Here, we conducted a large-scale epidemiological and genomic analysis of 5,895 *E. faecalis* genomes and identified ST179 as an emerging epidemic clone in China. Spot-killing assays demonstrated that ST179 exhibits strong inhibitory activity against several clinical *E. faecalis* STs. Biochemical purification coupled with proteomic analyses pinpointed BacL1 as a key effector associated with this antibacterial activity, and functional assays further supported its contribution to the observed inhibitory phenotype. Together, these findings show that the acquisition of the Bac41 operon may provide ST179 with a lineage-specific bacteriocin-mediated competitive advantage, likely contributing to its emergence and ecological success.

## RESULTS

### Global emergence of the *E. faecalis* lineage ST179

**Fig 1:**
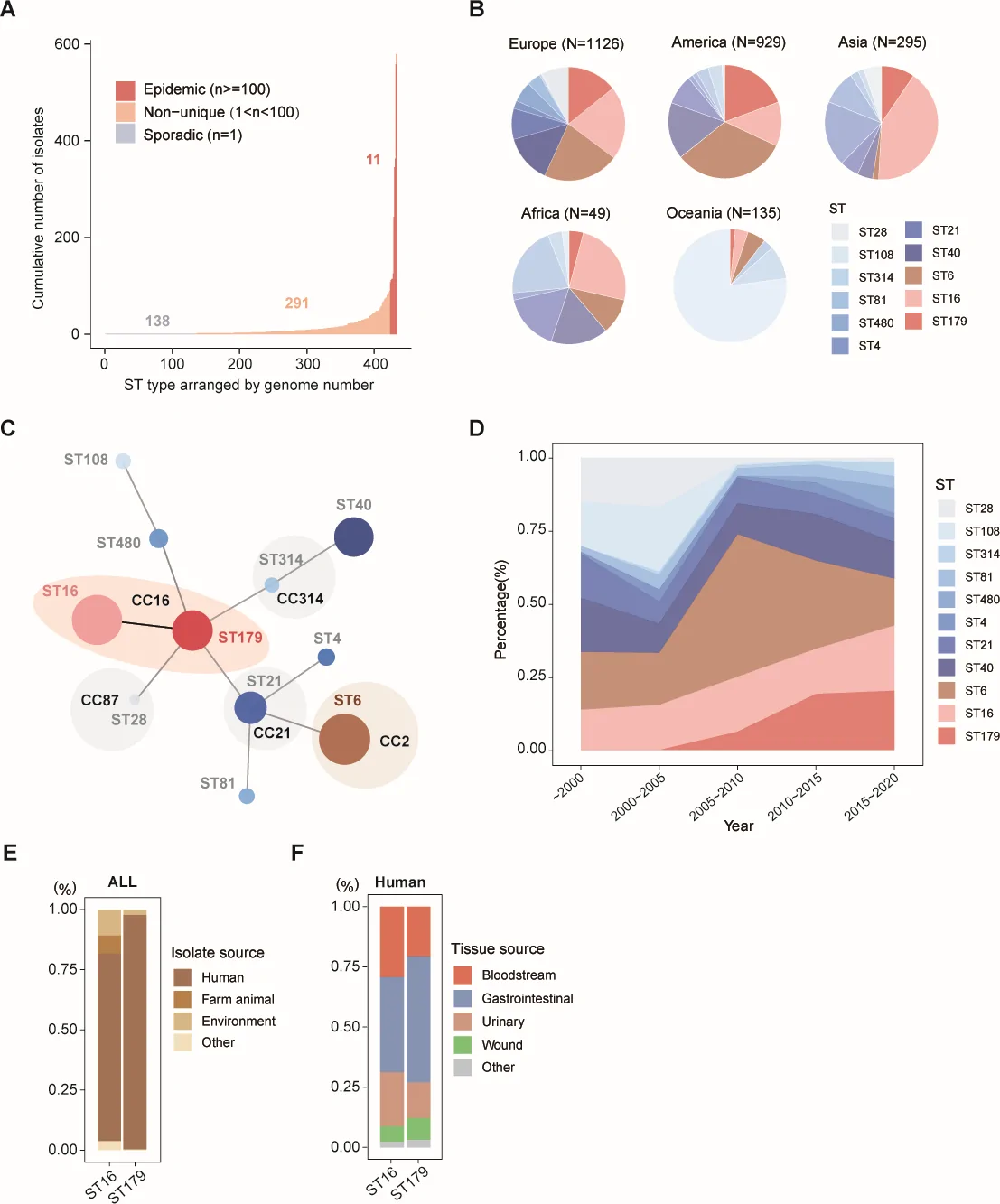
Population structure and temporal distribution of 5,895 *E. faecalis* isolates. (**A**) Cumulative number of isolates categorized by frequency of occurrence: epidemic (*n* = 100), non-unique (1 < *n* < 100), and sporadic (*n* = 1). ST types are arranged by the number of isolates. (**B**) Geographic distribution of the major STs across different continents. The bar length represents the relative abundance of each ST within the specified region. (**C**) A minimum spanning tree based on core-genome MLST profiles shows the genetic distance and clustering patterns of major STs and their associated clonal complexes (CCs). (**D**) Temporal trends in the relative prevalence (percentage of total annual isolates) of the major STs from approximately 2000 to 2020. (**E**) Distribution of isolation sources for the lineages ST16 and ST179. (**F**) Distribution of specific tissue sources for human-derived isolates of ST16 and ST179.

A total of 5,895 *E. faecalis* genomes collected between 2000 and 2020, including 5,665 publicly available genomes and 230 newly sequenced clinical isolates, were analyzed (Data set S1). Multilocus sequence typing (MLST) identified more than 400 distinct sequence types, which were categorized by frequency into epidemic (≥100 isolates), non-unique (1 < *n* < 100), and sporadic (*n* = 1) groups (Fig. 1A). Most isolates were sporadic, whereas 11 STs qualified as epidemic and collectively constituted the dominant global population.

The isolates originated from Europe (*n* = 1,126, 19.1%), America (*n* = 929, 15.8%), Asia (*n* = 295, 5.0%), Oceania (*n* = 135, 2.3%), and Africa (*n* = 49, 0.8%) (Fig. 1B). These epidemic STs exhibited marked geographic structure: ST16 and ST179 were prevalent across Europe, America, Asia, and Africa; ST108 was enriched in Oceania; and ST6 occurred frequently in Europe and America but was nearly absent in Asia. A minimum-spanning tree based on core-genome single nucleotide polymorphisms (SNPs) resolved several major clonal complexes (CCs), including CC16, CC2, CC21, CC87, and CC314, with CC16 and CC2 forming the largest clusters (Fig. 1C).

To investigate temporal population dynamics, we examined the relative prevalence of major STs from 2000 to 2020 (Fig. 1D). Prior to 2005, ST28, ST108, ST6, and ST16 dominated, collectively accounting for 63.8% of all isolates. ST179 first emerged in 2006 (2.8% of isolates) and expanded rapidly to 8.3% by 2010, coinciding with the disappearance of ST28 and ST108. From 2005 onward, ST179 continued its upward trajectory, reaching 13.7% by 2020, while ST6 declined to 9.9%. These trends indicate that ST179 has recently established itself as a globally disseminated epidemic lineage. Consistent with this global pattern, multiple regional studies across China over the past decade have reported ST179 as an increasingly prevalent hospital-associated clone (Table S1).

Concomitant with its expansion, ST179 displayed ecological patterns distinct from its close relative ST16 within CC16. ST179 isolates were overwhelmingly human-derived (96.8%), whereas ST16 was found across human (76.6%), animal (7.4%), and environmental (10.6%) sources (Fig. 1E). Furthermore, ST179 was frequently isolated from gastrointestinal samples (50.3%) (Fig. 1F).

### ST179 display an intraspecies advantage over clinical *E. faecalis* STs

ST16 and ST179 both belong to the CC16 clonal complex, and comparative genomics (ST16, *n* = 56; ST179, *n* = 27) showed that the two STs share broadly similar genomic features, including genome size (ST16, 2.91 ± 0.33 Mb; ST179, 3.06 ± 0.74 Mb) and GC content (ST16, 37.27% ± 0.18%; ST179, 37.42% ± 1.01%) (Fig. S1A and B) but differ in their virulence and antimicrobial resistance gene profiles. Notably, ST179 strains more frequently carried virulence genes associated with biofilm formation and exoenzyme production compared to ST16 strains (Fig. S1D). In addition, ST16 preferentially possessed multiple antibiotic resistance genes associated with the LSA phenotype, lincosamides, trimethoprim, and phenicols (Fig. S1C). Despite these genomic differences, the two lineages exhibited comparable growth curves and serum resistance (Fig. S1E and F). However, paired spot-killing assays revealed a marked difference: ST179 consistently produced clear inhibitory zones, whereas most ST16 isolates showed little or no detectable killing activity (Fig. S1G).

**Fig 2:**
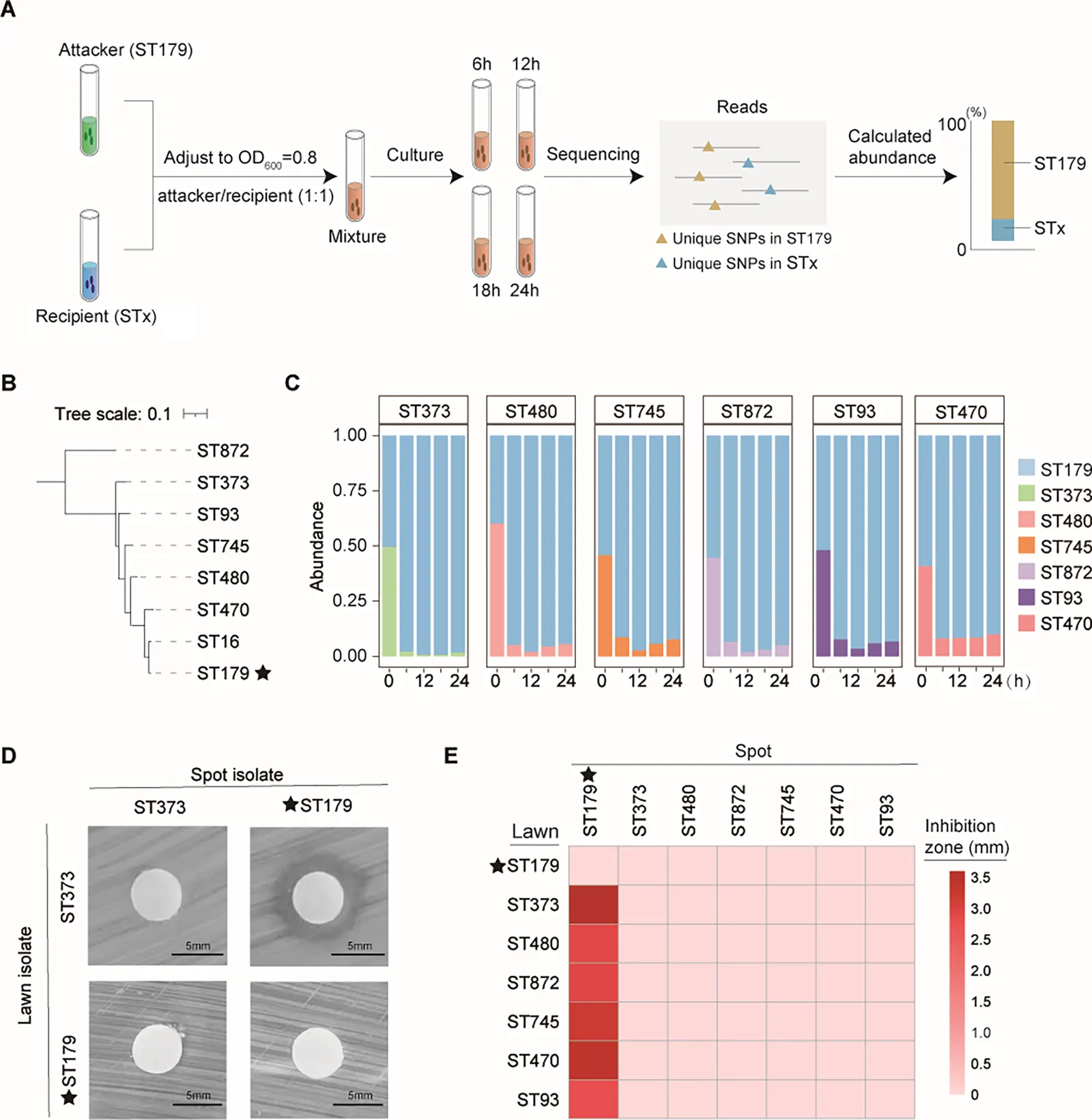
Competitive interactions of ST179 with clinical *E. faecalis* STs. (**A**) Schematic overview of the liquid killing assay. Attacker strain ST179 and recipient strains were adjusted to OD_600_ = 0.8, mixed at a 1:1 ratio, and incubated for 6, 12, 18, and 24 h at 37°C with shaking (220 rpm). (**B**) Maximum-likelihood phylogenetic tree of eight clinical *E. faecalis* isolates representing different sequence types, based on whole-genome SNPs. Scale bar indicates relative branch lengths. (**C**) Relative abundance of ST179 and six recipient strains during the liquid killing assay, calculated from SNPs of seven housekeeping genes for each ST. (**D**) Spot-killing assay showing inhibition of the recipient strain ST373 by ST179. (**E**) Quantification of inhibition zones (mm) from spot-killing assays of ST179 against six recipient strains (ST373, ST480, ST745, ST872, ST93, and ST470). Values represent the mean of three independent experiments.

To further assess the intraspecies competitive advantage of ST179, we performed a liquid killing assay using ST179 as the attacker strain and six representative clinical ST isolates, including ST373, ST480, ST745, ST872, ST93, and ST470, as recipients (Fig. 2A). In this co-culture system, ST179 was mixed 1:1 with each isolate. Quantification of strain-specific SNPs showed that ST179 progressively increased in relative abundance during the 6–24 h co-culture period, with the sharpest rise occurring within the first 6 h (Fig. 2A through C; Fig. S1E). These data demonstrate that ST179 inhibits the growth of diverse clinical STs under mixed-culture conditions. Phylogenetic analysis confirmed that ST179 is genetically distinct from the tested STs (Fig. 2B), indicating that its inhibitory phenotype is lineage specific.

We next performed pairwise spot-killing assays between ST179 and the same seven clinical isolates. Consistent with the liquid co-culture results, ST179 produced visible inhibition zones against all strains (Fig. 2D). The strongest inhibition was observed against ST373, with moderate but reproducible inhibitory effects against ST480, ST745, ST872, ST93, ST470, and ST16 (Fig. 2D; Fig. S1E).

Together, these findings demonstrate that ST179 possesses robust inhibitory activity against multiple clinical *E. faecalis* STs, likely contributing to its recent epidemiological emergence and expansion.

### Acquired Bac41 operon contributes to the intraspecies advantage of ST179

**Fig 3:**
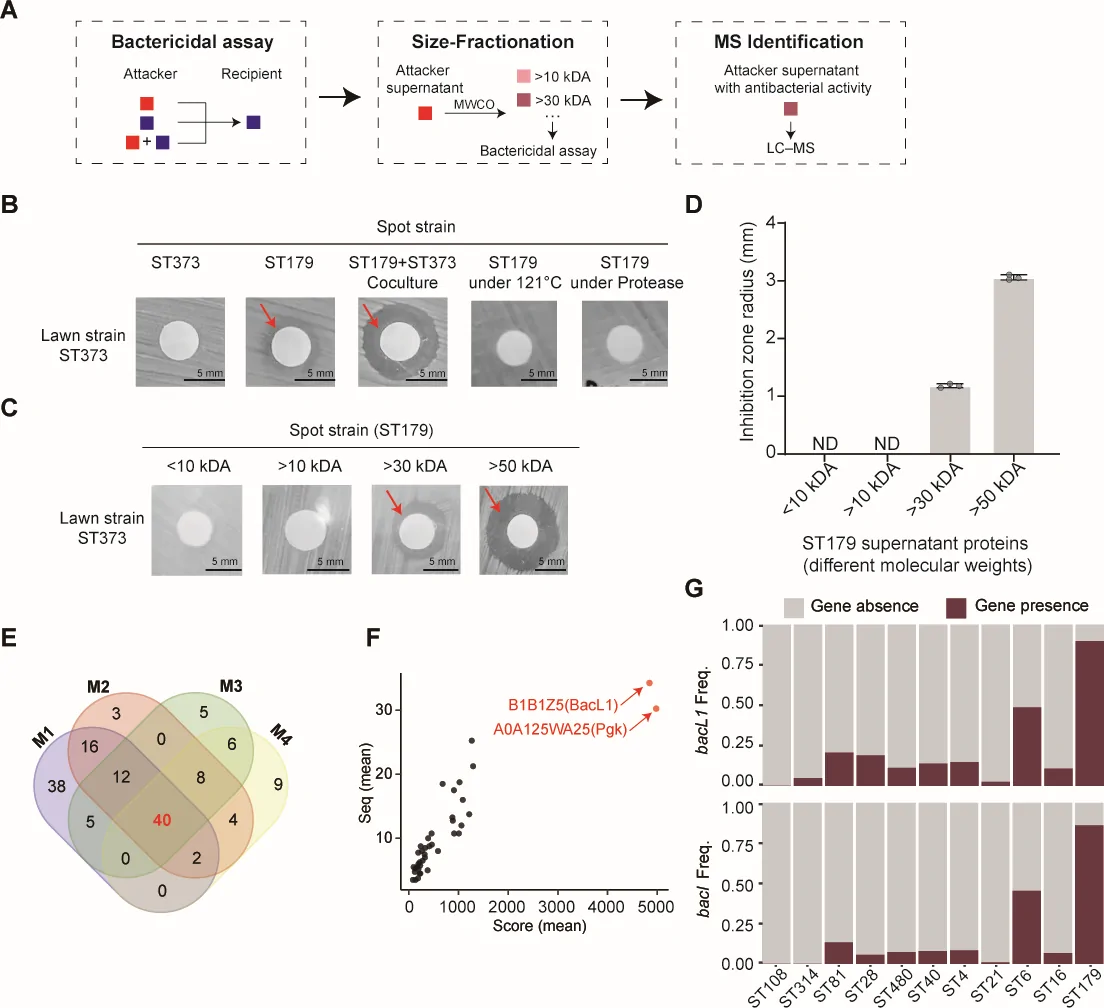
Screening and identification for antibacterial proteins in ST179. (**A**) A schematic diagram of the overall workflow to identify antibacterial proteins from the active supernatant of ST179. (**B**) Spot-killing assay showing the antibacterial activity of bacterial supernatants from *E. faecalis* ST179, ST373, their co-culture, and ST179 subjected to heat inactivation (121°C) or protease digestion against the recipient strain ST373. The red arrow indicates the inhibition zone. (**C**) Spot-killing assay showing the antibacterial activity of different size-fractionated bacterial supernatants of attacker strain ST179 against the recipient strain ST373. The red arrow indicates the inhibition zone. (**D**) Spot-killing assay result of different size-fractionated bacterial supernatants of attacker strain ST179 against the recipient strain ST373. The inhibition zone values are presented as mean ± SD from three biological replicates. (**E**) Venn diagram of proteins in the >30 kDa ST179 supernatant fraction. The diagram depicts the overlap of proteins identified by LC-MS/MS across four biological replicates (M1–M4). The core set of proteins (*N* = 40), conserved across all replicates, is highlighted in red. (**F**) Characterization of the core protein set identified by LC-MS/MS. Scatter plot of the 40 core proteins shows the relationship between the mean protein score (*X*-axis) and the mean number of significant unique peptides (*Y*-axis) per protein. Two proteins are highlighted in red. (**G**) Abundance of *bacL1* and *bacI* genes within major epidemic STs.

To identify the factors underlying the intraspecies competitive advantage of ST179, we integrated spot-killing assays, molecular weight cut-off (MWCO) fractionation, and LC-MS/MS proteomic analysis to screen for candidate proteins (Fig. 3A).

We first performed spot-killing assays using ST179 as the attacker and ST373 as the representative recipient. The culture supernatant of ST179 produced a clear, reproducible inhibition zone on the ST373 lawn, whereas the ST373 supernatant showed no activity (Fig. 3B), indicating that the inhibitory factor is secreted by ST179. Notably, the co-culture supernatant of ST179 and ST373 generated an even larger inhibition zone than ST179 alone, suggesting that interstrain interactions may enhance production or release of the active antibacterial molecule (Fig. 3B).

To characterize the nature of the inhibitory factor, we subjected the ST179 supernatant to heat treatment (121°C) or protease digestion. Both treatments abolished activity against ST373 (Fig. 3B), indicating that the effector is proteinaceous. MWCO fractionation revealed that inhibitory activity was restricted to the >30 kDa fraction (Fig. 3C and D), identifying the effector as a high-molecular-weight protein.

Proteomic analysis of the active >30 kDa fraction by LC-MS/MS identified 40 proteins consistently detected across four biological replicates (Fig. 3E). Among these, two proteins exhibited both high scores and multiple unique peptides (Fig. 3F). One was annotated as a bacteriocin-like protein encoded by *bacL1*, with 99.8% sequence identity to *bacL1* of Bac41 operon from plasmid pY114 (Fig. S2C; Table S2); the other corresponded to the housekeeping gene *pgk*, which is essential for glycolysis and unlikely to mediate antibacterial activity.

To determine whether *bacL1* is enriched in epidemic lineages, we screened major STs for the presence of *bacL1*. The gene was detected in 89.8% of ST179 isolates but was rare or absent in other STs, including ST16 (Fig. 3G). Its cognate immunity gene *bacI* showed a similar pattern, present in 86.0% of ST179 isolates but in only 6.7% of ST16 isolates. Besides, the Bac41 operon in clinical *E. faecalis* genomes showed two distinct organization patterns compared with plasmid pY114. These patterns were designated as complete Bac41 operon and partial Bac41 operon based on the presence of all four genes (*bacL1*, *bacL2*, *bacA*, and *bacI*) (Fig. S2A). Among clinical strains, ST179 genomes showed the highest proportion of complete Bac41 operon (80.8%) (Fig. S2B). These results suggest that the Bac41 operon is enriched in the ST179 lineage and may contribute to its intraspecies competitive advantage, with BacL1 acting as a representative effector protein.

### Bac41 operon is associated with intraspecies antibacterial activity in ST179

**Fig 4:**
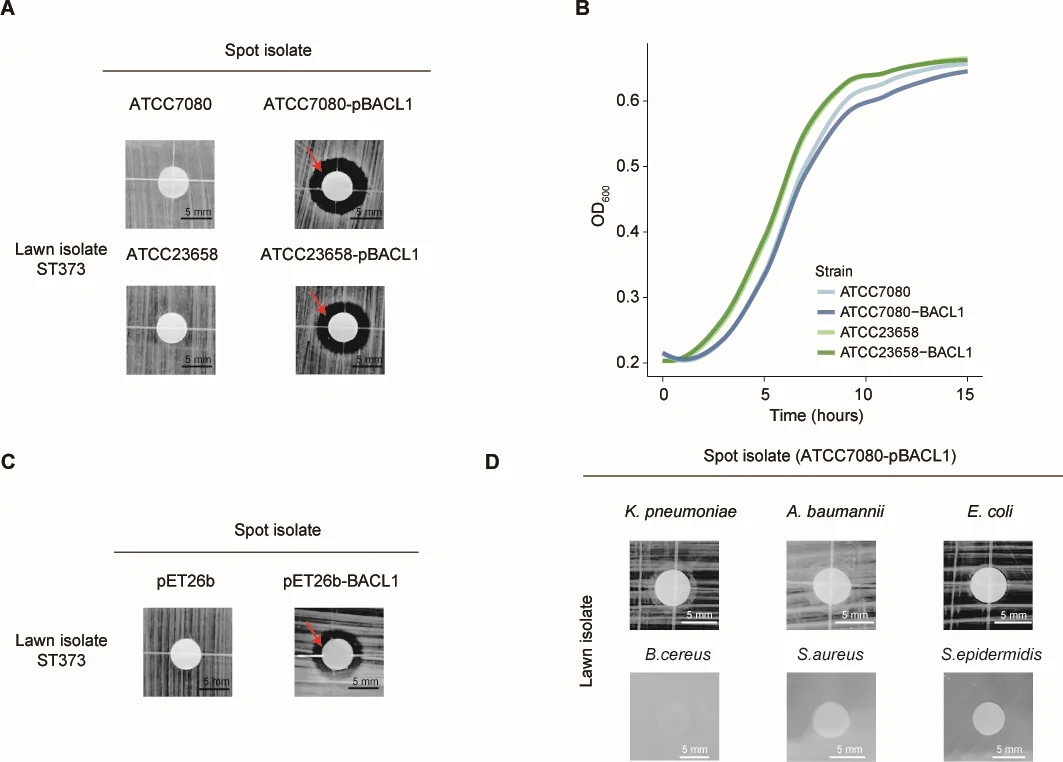
Functional validation of BacL1 as the key antibacterial effector in *E. faecalis*. (**A**) Spot-killing assays showing the antibacterial activity of culture supernatants from *E. faecalis* ATCC 7080 and ATCC 23658 strains carrying either the empty vector or the *bacL1* expression plasmid (pBACL1) against the recipient strain ST373. The red arrow indicates the inhibition zone. (**B**) Growth curves of *E. faecalis* ATCC 7080 and ATCC 23658 carrying the empty vector or pBacL1 cultured at 37°C. Data represent the mean OD_600_ values from three biological replicates. (**C**) Spot inhibition assay showing the antibacterial activity of affinity-purified recombinant His-tagged BacL1 expressed in *Escherichia coli* from pET26b-BACL1 against the *E. faecalis* strain ST373. Material prepared in parallel from *E. coli* carrying the empty vector pET26b was used as the negative control. The red arrow indicates the inhibition zone. (**D**) Spot-killing assays showing the antibacterial activity of culture supernatants from *E. faecalis* ATCC 7080 carrying pBACL1 against strains of other species.

To determine whether the Bac41 operon-encoded BacL1 contributes to the intraspecies antibacterial activity of *E. faecalis* ST179, we expressed *bacL1* in two non-inhibitory reference strains, ATCC 7080 and ATCC 23658, neither of which naturally carries the gene. PCR and immunoblotting confirmed successful plasmid construction and gene expression. Both recombinant strains harboring *bacL1* produced clear inhibition zones against the recipient strain ST373, whereas wild-type strains containing empty vectors showed no activity (Fig. 4A). These results indicate that heterologous expression of *bacL1* was sufficient to confer antibacterial activity in *E. faecalis* strains lacking the gene.

To directly assess the role of BacL1 in bactericidal activity, we constructed a nisin-inducible expression system in *E. faecalis* ATCC 23658, which does not naturally carry *bacL1*. Induction with increasing concentrations of nisin led to enhanced BacL1 accumulation and a corresponding significant enlargement of inhibition zones against indicator *E. faecalis* strains (Fig. S2D). These results indicate that BacL1 expression level correlates positively with bactericidal activity. The inhibitory phenotype was not attributable to growth defects or plasmid burden, as *bacL1*-expressing strains and vector controls displayed comparable growth kinetics under standard culture conditions (Fig. 4B).

To determine whether BacL1 protein alone is sufficient to exert antibacterial activity, recombinant BacL1 was heterologously expressed and purified. The purified recombinant BacL1 exhibited strong inhibition against *E. faecalis* ST373, whereas control preparations had no effect (Fig. 4C). Spot-killing assays using culture supernatants from *E. faecalis* ATCC 7080 expressing *bacL1* showed no inhibition against heterologous bacterial species, including *Klebsiella pneumoniae*, *Acinetobacter baumannii*, *E. coli*, *Staphylococcus aureus*, *Staphylococcus epidermidis*, and *Bacillus cereus*, indicating that BacL1 activity is species specific (Fig. 4D).

Together, these findings suggest that the Bac41 operon is associated with intraspecies antibacterial activity in *E. faecalis* ST179.

## DISCUSSION

To elucidate the evolutionary mechanisms underlying the emergence of *E. faecalis* lineages, we investigated the genomic characteristics and biological functions of the epidemic high-risk clone ST179 (3). Our analyses reveal that ST179 possesses unique competitive traits that likely account for its rapid epidemiological expansion across diverse geographic and clinical contexts (9, 23, 29). Among these traits, we identified the acquisition of bacteriocin Bac41 that appears to contribute to the competitive advantage and dissemination of ST179 (26).

Bac41 was originally identified on plasmid pY114 in the clinical strain *E. faecalis* YII4 (30). It encodes a multicomponent bacteriocin system in which BacL1 and BacA represent key functional effectors. BacL1 functions as an endopeptidase whose activity depends on BacA, while BacI provides self-immunity to the producing strain by preventing autotoxicity (26, 30, 31). In contrast, our findings demonstrate that BacL1 exhibits detectable antibacterial activity against *E. faecalis* in heterologous expression and purified protein assays, supporting its intrinsic bactericidal potential. This does not imply that BacL1 functions independently in the native Bac41 system, but rather that its activity may be modulated by regulatory or complex-associated constraints in its physiological context.

The BacI immunity factor is widely present in ST179 and protects against self-targeted killing. BacI also exhibits subtype diversity, suggesting variability in immunity mechanisms (26). Interestingly, some clinical strains carrying *bacL1* but lacking *bacI* are naturally present and still survive, which may reflect subtype-specific immunity rather than true absence of immunity function (22). Collectively, these observations indicate that Bac41-associated competition is influenced not only by gene presence or absence but also by BacI subtype diversity.

We were unable to generate a *bacL1* knockout mutant in clinical ST179 isolates due to their genetic intractability, which precluded direct assessment of its essentiality in intraspecies competition. Therefore, inducible expression and purified protein assays were used as complementary approaches. BacL1 exhibited dose-dependent inhibitory activity, and the effects observed in the inducible system were not attributable to nisin, consistent with established non-inhibitory concentrations in *E. faecalis* (32–35). Together, these results, supported by the *in vitro* bactericidal activity of recombinant BacL1, suggest a contributory role of BacL1 in the competitive phenotype. These findings indicate that Bac41-associated functions may vary depending on the genetic background of *E. faecalis* lineages.

ST179 is a human-adapted, single-operon variant within the CC16 clonal complex and has recently emerged as a clinically important lineage in multiple regions, including China, Japan, Saudi Arabia, and the United States, where it is frequently associated with urinary tract infections and infective endocarditis (3, 9). Recent studies from China report that compared with ST16, ST179 exhibits slightly reduced biofilm formation but carries a broader set of virulence determinants and multidrug resistance genes (36). However, these features alone do not fully explain its epidemiological success.

An intriguing finding of our study is that the Bac41 operon-encoded BacL1 protein is strongly associated with the emerging ST179 clone. It should be noted that it is also present in a subset (10.5%) of ST16 isolates. Therefore, we confirmed that *bacL1*-positive ST16 isolates possess functional bactericidal activity comparable to ST179 *in vitro*. This indicates that the presence of a functional *bacL1* gene alone is not sufficient to guarantee a strain’s epidemiological success.

We propose that the impact of the Bac41 operon on strain prevalence should be modulated by the broader genetic context and ecological niche of the host strain. Our data show that ST179 is more highly adapted to the human gut niche, the primary site of inter-strain competition. In contrast, ST16 is isolated from both human and environmental sources. Furthermore, ST179 carries a large number of virulence factors, while ST16 is enriched with antimicrobial resistance genes. We hypothesize that in ST16, the fitness cost associated with maintaining a large antimicrobial resistance (AMR) gene repertoire, combined with its suboptimal adaptation to the human gut, may negate the competitive advantage provided by Bac41. Conversely, ST179, which is well adapted to the gut niche and carries a lower AMR-associated burden, can effectively leverage BacL1 to outcompete other enterococci, including ST16, thereby driving its rapid expansion as an epidemic clone in China.

In conclusion, our study shows that the Bac41 operon is highly prevalent in the epidemic *E. faecalis* clone ST179 and is associated with a lineage-specific intraspecies competitive advantage, which may partly explain its emergence and dissemination. From an epidemiological standpoint, this feature raises concern that ST179 could continue to evolve into a high-risk global clone, particularly in China. Continued large-scale genomic analyses and sustained epidemiological surveillance will be essential for tracking the spread of ST179 and for informing strategies to anticipate, control, and mitigate the expansion of this potentially high-risk lineage.

## MATERIALS AND METHODS

### Genome collection

Five thousand six hundred sixty-five publicly available *E. faecalis* genome sequences covering the period from approximately 2000 to 2020 were obtained from the NCBI GenBank and ENA databases. These genomes have clearly documented collection dates, collection regions, and assigned sequence types and were used in downstream analyses. In addition, 230 clinical *E. faecalis* isolates were obtained from the clinical laboratory of Beijing Ditan Hospital (2011–2014) and China-Japan Friendship Hospital (2004–2015). These genomic data of isolates collected from Beijing Ditan Hospital have been deposited in the National Genomics Data Center (NGDC; https://ngdc.cncb.ac.cn/) under BioProject accession number PRJCA006264.

### Genome sequencing, assembly, and annotation

Public *E. faecalis* genomes were downloaded and annotated using Prokka (37). Genome sequence files were processed with BioPerl (https://bioperl.org/) for downstream genome-wide analyses. The clinical isolates were cultured, and total genomic DNA was extracted using a genomic DNA kit (Qiagen, USA) following the manufacturer’s instructions. Sequencing libraries were prepared using the NEBNext Ultra DNA Library Prep Kit for Illumina (NEB, USA), and whole-genome sequencing was performed on the Illumina NovaSeq platform. Raw reads were quality checked using FastQC, and paired-end reads were assembled *de novo* with SPAdes. The resulting assemblies were annotated using Prokka (38, 39).

### Multilocus sequence typing

MLST profiles of all *E. faecalis* genomes were determined using the MLST pipeline with default parameters (https://github.com/tseemann/mlst) (40). Genome sequences were compared to the nucleotide sequences of seven housekeeping genes (*gdh*, *gyd*, *pstS*, *gki*, *aroE*, *xpt*, and *yqiL*) in the MLST database (https://pubmlst.org/organisms/enterococcus-faecalis) to identify alleles and assign STs. Genetic relationships among genomes were visualized by constructing a minimum spanning tree using Phyloviz (41).

### Phylogenetic analysis

Seven clinical *E. faecalis* genomes were mapped to the reference genome of *E. faecalis* strain V583 (GenBank accession number AE016830) using Snippy to identify SNPs. The identified variant sites were concatenated and aligned across all strains with MUSCLE (42). Phylogenetic reconstruction was conducted using FastTree based on the maximum-likelihood method. The resulting tree was visualized and annotated with iTOL (43, 44).

### Identification of virulence and antibiotic resistance genes

A BLASTN search was conducted using all predicted genes from the *E. faecalis* genomes against VFDB and ResFinder databases to identify putative virulence and antibiotic resistance genes (45–47). Hits with an *E*-value ≤1e−5, nucleotide identity ≥75%, and alignment coverage ≥75% were considered positive.

### Identification of the Bac41 operon

A BLASTN search was conducted using all predicted genes from the *E. faecalis* genomes against the reference Bac41 operon (30) (GenBank accession number AB271686.1) to identify putative *bacL1*, *bacL2*, *bacA,* and *bacI* homologs. Hits with an E-value ≤1e−5, nucleotide identity ≥75%, and alignment coverage ≥75% were considered positive. The organization of the Bac41 operon was illustrated using a custom Perl script. Multiple sequence alignments of BacL1 protein sequences were performed using MUSCLE (42) and visualized with ESPript 3.0 (48).

### Liquid competition assay

Eight *E. faecalis* sequence types (ST179, ST16, ST93, ST373, ST470, ST480, ST745, and ST872) were revived from frozen stocks and cultured overnight. Each strain was adjusted to 1 × 10⁷ CFU mL^−1^. ST179 was competed individually against each of the other seven sequence types by mixing equal volumes (1 mL each) of the two strains. The mixtures were inoculated into 100 mL of fresh brain heart infusion (BHI) broth in Erlenmeyer flasks and incubated at 37°C with shaking at 220 rpm. Samples (1 mL) were collected at 0, 6, 12, 18, and 24 h for genomic DNA extraction (23, 29). Whole-genome sequencing of these samples was performed, and SNP-based analysis of seven housekeeping genes was used to determine genomic variation and the relative abundance of each strain over time.

### Spot-killing assay

Test strains were cultivated for 16–18 h at 37°C in brain heart infusion broth. Subsequently, 5 mL of a BHI top agar lawn (containing 0.35% agar) was prepared by mixing the molten agar with 100 μL of a 1:100 dilution of the overnight culture. This mixture was poured on top of 25 mL of solid BHI agar. Competing strains were spotted (5 μL undiluted overnight culture) onto solidified top agar lawns. Inhibition zones were measured (in mm) after 16–18 h of incubation at 37°C (29).

### Cloning and expression of bacteriocin BacL1

The bacteriocin BacL1 was cloned into the expression vector pNZ8148, which was modified to carry the chloramphenicol resistance gene *cat* as a selectable marker (49, 50). The insert was amplified from *E. faecalis* DT4040 genomic DNA by PCR using primers 5′-ATG AAT TAC AGT CAA AAA GC-3′ and 5′-CTA ATT AAA GAA TCC TTT GC-3′. The vector was amplified by PCR using primers 5′-GCA AAG GAT TCT TTA ATT AGA GAG CTC AAG CTT TCT TTGA-3′ and 5′-GCT TTT TGA CTG TAA TTC ATG TAC CCA TGG TGA GTG CCTC-3′. The purified insert and vector were assembled via Gibson assembly (HiFi DNA Assembly Cloning Kit, NEB) and transformed into NEB 5-alpha competent *E. coli*. Transformants were selected on BHI agar supplemented with 10 µg mL^−1^ chloramphenicol. Plasmids were amplified in 200 mL cultures, purified by Maxiprep, and sequenced to confirm identity. The BacL1-encoding vector (pBACL1) or the empty pNZ8148 vector was subsequently transformed into the BacL1-negative *E. faecalis* strain ATCC 7080. Successful transformation was verified by PCR using pBacL1-specific primers. The antibacterial activity of ATCC 7080 carrying pBACL1 was confirmed in a pairwise spot-killing assay against ATCC 7080 transformed with the empty vector.

### Nisin-induced expression of BacL1 in *E. faecalis*

Overnight cultures were diluted 1:100 into fresh BHI broth and grown to the mid-log phase (OD_600_ = 0.4–0.6). Nisin stock solution was prepared in 0.02 M HCl, filter-sterilized through a 0.22 µm membrane, and added to the cultures at final concentrations of 0.25, 2.5, or 25 ng mL^−1^ (33, 49, 50). Control cultures received an equal volume of solvent without nisin. After 6 h of induction at 37°C, cultures were collected and used for downstream antibacterial assays.

### Expression and purification of recombinant BacL1

For recombinant protein production, *bacL1* was cloned into pET26b to generate pET26b-BacL1 expressing His-tagged BacL1 (31). The empty pET26b vector was used as a negative control. The plasmids were transformed into *E. coli* BL21, and transformants were selected on LB agar supplemented with 30 µg mL^−1^ kanamycin. A single colony was inoculated into LB broth containing 30 µg mL^−1^ kanamycin and grown at 37°C with shaking. Cultures were grown to an OD_600_ of approximately 0.8, and protein expression was then induced with IPTG at a final concentration of 0.5 mM. After induction, cultures were incubated at 25°C for 4 h.

Cells were harvested by centrifugation, resuspended in phosphate-buffered saline (PBS), and lysed by sonication. The lysate was clarified by centrifugation, and the supernatant was applied to a Ni-NTA affinity column. Recombinant His-tagged BacL1 was eluted with PBS containing 250 mM imidazole. The eluted protein was then exchanged into PBS by ultrafiltration to remove imidazole. Material prepared in parallel from *E. coli* BL21 carrying the empty pET26b vector and purified using the same procedure was used as the negative control. The antibacterial activity of purified BacL1 was assessed by spot inhibition assay against *E. faecalis* ST373.

### Serum bactericidal assay

The *E. faecalis* strains were cultured at 37°C in brain heart infusion medium (Oxoid Ltd., UK). The overnight bacterial cultures were diluted with fresh BHI medium to adjust the concentration to 1 × 10^6^ CFU/mL (optical density at 600 nm [OD_600_] = 1). For the serum bactericidal assay, 2 × 10^5^ CFU (25 μL) of the *E. faecalis* strains was mixed with 75 μL normal human serum, and the mixture was then incubated at 37°C. This mixture suspension was made in triplicate. Next, 100-fold serial dilutions from each suspension were obtained at 0, 1, 3, and 6 h, respectively, and then coated on BHI medium. Surviving bacteria were quantified by counting the CFU after incubation at 37°C for 24 h. Each experiment was replicated three times.

### Statistical analysis

Nonparametric data were analyzed by the Kruskal-Wallis test or Wilcoxon test in the “ggpubr” package (https://CRAN.R-project.org/package=ggpubr) in R (v4.1.2, https://www.r-project.org/), depending on the number of variables. *P-*values ≤0.05 were considered statistically significant (0.01 ≤ *P* < 0.05, *; 0.001 ≤ *P* < 0.01, **; 0.0001 ≤ *P* < 0.001, ***; and *P* < 0.0001, ****).

## SUPPLEMENTAL MATERIAL

Data Set S1 spectrum.03982-25-s0001.xlsx Global epidemiological studies of *E. faecalis* clonal complex 16.

Supplemental material spectrum.03982-25-s0002.docx Fig. S1 and S2; Tables S1 and S2.

ASM does not own the copyrights to Supplemental Material that may be linked to, or accessed through, an article. The authors have granted ASM a non-exclusive, world-wide license to publish the Supplemental Material files. Please contact the corresponding author directly for reuse.
