# Supplementary material for: Bacteriocin-mediated intraspecies competition driven by acquired Bac41 operon in epidemic Enterococcus faecalis ST179
Source: Microbiol Spectr. 2026 Aug 6;14(9):e03982-25. doi: 10.1128/spectrum.03982-25 (PMC13532309; doi:10.1128/spectrum.03982-25)
Supplement: Supplementary file 1 [file spectrum.03982-25-s0002.docx]

**Supplemental Figures and Tables**

**Figure S1. Comparative genomic and phenotypic characteristics of clinical *E. faecalis* ST16 and ST179 isolates.**

A. Genome size (Mb) of ST16 and ST179 isolates.
B. Genome GC content (%) of ST16 and ST179 isolates.
C. Heatmap showing the distribution of virulence genes between ST16 and ST179 isolates.
D. Heatmap showing the distribution of antibiotic resistance genes between ST16 and ST179 isolates.
E. Growth curves of ST16 and ST179 isolates.
F. Serum bactericidal ability of ST16 and ST179 isolates.
G. Paired spot killing assays within ST16 (*bacL1*^-^)、ST16 (*bacL1*^+^) and ST179 isolates. The red arrow indicates the inhibition zone.

**Figure S2. Organization and distribution of the Bac41 operon in *E. faecalis*.**

A. Schematic organization of the Bac41 operon in plasmid pYI14 and representative complete or partial Bac41 loci identified in clinical *E. faecalis* isolates. Arrows with colors indicate coding sequences, including *bacL1*, *bacL2*, *bacA*, and *bacI*.
B. Proportions of clinical *E. faecalis* isolates carrying complete Bac41 operon, partial Bac41 operon, or no Bac41 operon across major sequence types.
C. Multiple sequence alignment of BacL1 from plasmid pYI14 and clinical ST179 strains. Conserved residues are highlighted in red, with predicted secondary structure shown above.
D. *E. faecalis* strains carrying PnisA-bacL1 were cultured in the presence of different concentrations of nisin. Antibacterial activity was evaluated by agar diffusion assay. The White dotted border outlines the extent of the inhibition zone.

**Supplemental Table 1. Global epidemiological studies of *E. faecalis* clonal complex 16 (CC16).**

| **PMID** | **Country** | **Collection year** | **CC16 frequency** | **Strain number** |
| --- | --- | --- | --- | --- |
| PMC11705914 | Shanghai, China | 2022-2023 | ST16 (33.8%),  ST179 (18.8%) | 80 |
| PMC12323598 | Sweden | 2020-2021 | -- | 194 |
| PMC11218351 | Zhejiang, China | 2012-2021 | -- | 1,224 |
| PMC9855154 | Northern Japan | 2022 | CC16 (48%) | 426 |
| PMC5705541 | Shenzhen, China | 2010-2016 | CC16 (65.6%) | 265 |
| 29923 823 | Single-center hospital, China | 2011-2014 | -- | 77 |
| PMC6434864 | Western Saudi Arabia | 2019 | -- | 155 |
| 22207599 | Multi-center hospital, Europe | 2006-2009 | -- | 386 |

**--** The study did not report the specific prevalence of CC16, but it indicated that CC16 was a major clone

**Supplemental Table 2. Sequence comparison of Bac41 operon genes in clinical genomes.**

| Bac41 operon Gene | Identity (%) | Coverage (%) |
| --- | --- | --- |
| *bacL1* | 87.5~99.9 | 100 |
| *bacL2* | 98.9~100 | 84.7~100 |
| *bacA* | 92.4~99.3 | 90.3~100 |
| *bacI* | 99.4~99.6 | 100 |
